# Theoretical framework and differentiated policies for national park zoning management: A Baishanzu case study in China

**DOI:** 10.1016/j.isci.2024.111377

**Published:** 2024-11-13

**Authors:** Yuchao Cai, Yingnan Zhang, Yuzhe Wu

**Affiliations:** 1 Department of Land Management, School of Public Affairs, Zhejiang University, Hangzhou 310058, P.R. China

**Keywords:** Environmental science, Environmental management, Environmental policy, Ecology

## Abstract

Human activities are contributing to a global decline in biodiversity and ecosystem services. While national parks, rooted in sustainable development principles, aim to counteract this trend and have been successful in developed nations, their direct applicability to developing countries is debatable. In light of the challenges associated with coordinating environmental, social, economic, and cultural objectives, we have formulated a theoretical framework centered on the concept of “risk-value” for managing national park zoning in developing nations. This framework is designed to promote co-prosperity by simultaneously addressing biodiversity conservation, equity, and human well-being concerns. Our framework, when applied to China’s Baishanzu National Park, entailed subdividing the park into four distinct zones, each managed by specific and tailored policies. Our research provides insights into the theoretical underpinnings of implementing the national park concept in developing countries and showcases effective strategies for enhancing ecological conservation in these regions.

## Highlights

• Theoretically analyzed national park zoning management for developing countries
• Zoning according to the ecosystem service value and ecological risk
• Developed flexible strategies for each zone based on quantitative results
• Focus on balancing ecological protection with economic development

## Graphical abstract

**Figure:**
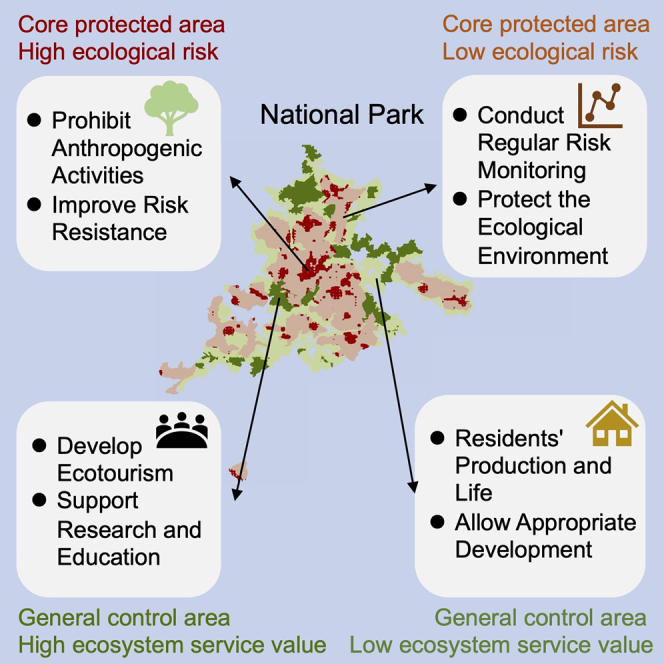

## Introduction

Anthropogenic pressures are having negative consequences for the ecosystems on which humans depend, and are causing a global decline of biodiversity and ecological service values.^1^^,^^2^^,^^3^ This decline is particularly worrisome given the critical role biodiversity plays in providing ecosystem services, ranging from food production and climate regulation to intrinsic cultural values.^4^ Consequently, the importance of biodiversity in maintaining ecosystem services cannot be overstated, and halting biodiversity loss is on the international agenda.^5^ The main direct drivers of current species declines include habitat loss, overexploitation, species invasions, and pollution.^6^^,^^7^ Protected areas mitigate these problems by limiting land use impacts and managing threats (e.g., invasive species) that may be ubiquitous outside of protected areas, thus playing a good role in conserving biodiversity.^8^^,^^9^ According to the IUCN classification system, national parks are an important type of protected area.^10^ Globally, national parks for conservation purposes are essential for the preservation of biodiversity and ecosystem functioning, and are increasingly recognized as central to biodiversity conservation.^11^^,^^12^^,^^13^^,^^14^^,^^15^^,^^16^ Currently, the world is threatened by an unprecedented decline in ecosystem services that are vital to human survival, while poverty is plaguing many parts of the world as well.^17^ In order to meet both conservation and utilization objectives, Sustainable Development Goals (SDGs)—ecologically sound economic growth—has become a practical necessity.^18^^,^^19^ Against this backdrop, the objectives of protected areas have diversified as they have continued to grow. Protected areas are now established not only to protect iconic landscapes and seascapes, to provide habitat for endangered wildlife and to play a key role in climate change mitigation and adaptation, but also to contribute to the livelihoods of local communities, to boost the national economy through tourism revenues, as well as to perform many other functions.^20^^,^^21^ Navigating biodiversity conservation, equity, and human well-being is crucial in the management of protected areas.^22^ National parks, as a form of protected area, aim to achieve multiple objectives, including natural resource conservation, equity, livelihoods, and the global SDGs.^23^

Despite their conservation goals, many national parks face challenges in species protection due to limited management practices.^24^ This issue is amplified by the diverse objectives within parks, which can create conflicts as stakeholders seek different uses of resources.^25^ Studies have shown that effective management strategies, particularly zoning (the allocation of land units to specific uses), can help mitigate conflicts and address threats like habitat loss.^26^^,^^27^^,^^28^^,^^29^^,^^30^^,^^31^ Zoning is an adaptable process, allowing for updates that respond to evolving conservation needs and resource demands, and is now widely used across both marine and terrestrial protected areas to prioritize ecological assets.^32^^,^^33^^,^^34^^,^^35^^,^^36^^,^^37^ By proactively identifying areas of ecological or cultural importance and areas of potential interest to specific sectors, these zoning schemes help reconcile the mechanisms for habitat and species conservation with sustainable human use, thereby minimizing conflicts between them. The zoning management of national parks in developed countries is mainly divided into two approaches: the concept of “wilderness”, exemplified by the United States, and the concept of “recreation”, exemplified by the United Kingdom.^38^ The concept of “wilderness” emphasizes preserving untouched natural ecosystems by imposing restrictions on human activities to maintain “nature-society” segregation and fostering a deeper appreciation and reverence for nature through wilderness encounters. Zoning under this concept focuses on varying wilderness characteristics, emphasizing the preservation of ecosystems largely untouched by modern civilization. Each zone offers opportunities for solitude or primitive, unconfined types of recreation that rely solely on human power.^39^^,^^40^ This approach is designed to conserve spectacular natural landscapes and wildlife, primarily in areas with little potential for economic utilization.^41^ Conversely, the concept of “recreation” is oriented toward human services, treating national parks as integral components of regional operation and development.^42^^,^^43^^,^^44^^,^^45^ It aims to enhance recreational opportunities and socio-economic welfare by protecting and enhancing natural landscapes with aesthetic, ecological, and cultural value.^46^ This concept is primarily implemented by countries such as the United Kingdom, which have smaller land areas and where natural environments are more heavily influenced by human activities. This concept assumes that plants and animals can thrive under natural, dynamic conditions and allows for the designation of specific zones to provide recreational and sporting facilities.^47^^,^^48^^,^^49^ It promotes the trend of commercializing and commodifying nature experiences, opening up national parks for recreational and tourism purposes to generate new opportunities for local socio-economic development and employment.^50^^,^^51^^,^^52^^,^^53^^,^^54^^,^^55^

Unarguably, the establishment of national parks has become one of the global trends toward the harmonization of nature conservation and human economic activities.^44^^,^^56^ The Convention on Biological Diversity (CBD) aims to protect 30% of critical biodiversity and ecosystem areas by 2030,^57^^,^^58^^,^^59^ and many developing countries, including China, have adopted a “core zone—buffer zone” model to balance ecological protection and sustainable development.^60^^,^^61^ However, in developing countries, park zoning often remains at the planning level, lacking specific management strategies for practical application. Several studies have highlighted the prevalence of poverty in most developing countries, leading to underfunding of national parks where revenues play a crucial role in their economies.^62^^,^^63^^,^^64^^,^^65^^,^^66^ Consequently, applying a “wilderness” approach—focused on ecosystem integrity and minimal human interaction—poses financial and operational challenges in these contexts. In such settings, conservation efforts risk stalling due to high implementation costs.^44^^,^^56^^,^^67^^,^^68^ Alternatively, directly applying national park management programs guided by the concept of “recreation”—which recognizes the interests and knowledge of local residents, empowers local communities and stakeholders to participate in park management and decision-making, and emphasizes the tourism attributes of national parks^69^^,^^70^ —to developing countries may lead to conflicting interests, contradictions, and ecological damage. Given these limitations, developing countries require tailored zoning strategies that prioritize both ecological goals and socioeconomic needs to achieve the global SDGs.

Landscape ecological risk reflects the potential for adverse impacts from human and natural factors, indicating an ecosystem’s resilience under environmental pressures.^71^^,^^72^^,^^73^^,^^74^^,^^75^^,^^76^ Assessing this risk often uses RS, GIS, and landscape metrics to construct an Ecological Risk Index (ERI), which integrates diverse disturbances and analyzes risk based on land cover changes.^77^ By focusing on spatial and temporal heterogeneity, ERI aids regional planning, landscape management, and resource allocation.^73^^,^^78^ Meanwhile, human activities, altering land cover types, impact ecosystem functions—such as provisioning, regulating, and cultural services—leading to shifts in Ecosystem Service Value (ESV).^79^^,^^80^^,^^81^^,^^82^^,^^83^^,^^84^ Scientific assessment of ESV is thus vital for quantifying ecosystem benefits and informs sustainable land use planning.^85^^,^^86^^,^^87^^,^^88^^,^^89^ ERI and ESV have been extensively applied across various landscapes, including river basins, protected areas, and urban regions, becoming core methods in ecological studies.^90^^,^^91^^,^^92^^,^^93^^,^^94^^,^^95^^,^^96^^,^^97^ Currently, the primary responsibilities of national parks are to balance environmental protection with economic development and to manage conflicts between differing objectives through zoning management. Individualized policies for different functional areas are crucial for achieving multiple objectives in national parks. For areas dedicated to ecological protection, the ERI is employed to assess their resilience to negative ecological impacts. For areas designated for human activities, the ESV is used to evaluate the potential for converting ecological values into economic benefits. This quantitative assessment provides valuable insights for formulating effective and scientifically grounded policies.

**Figure 1:**
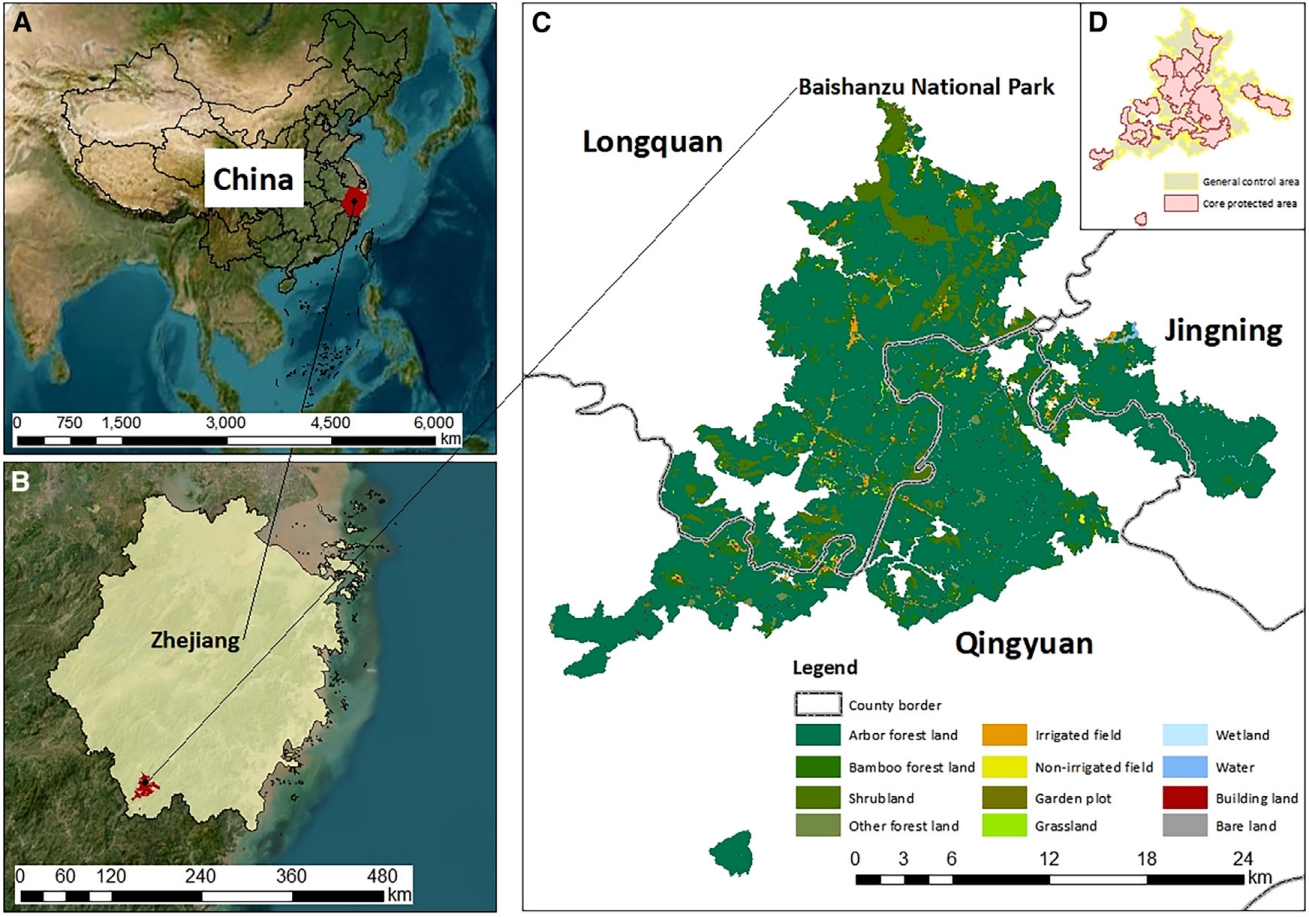
Location map of the Baishanzu National Park (A) Location of Zhejiang Province in China. (B) Location of the Baishanzu National Park in Zhejiang Province. (C) Land use and administrative districts in the Baishanzu National Park. (D) Zoning in the Baishanzu National Park. The maps were generated by ArcGIS 10.7.

China, with its large population and abundant natural resources, is a typical representative of developing countries. Meanwhile, the Chinese government is actively developing a network of nature reserves with a focus on national parks, and officially declared the establishment of the inaugural series of national parks in 2021. Against this backdrop, our case study area, Baishanzu National Park, was established in Zhejiang Province in southeastern China (Figure 1). This park is characterized by a typical forest ecosystem, boasting rich internal biological resources, diverse vegetation types, and a high concentration of endangered species. Additionally, during the initial establishment of this national park, the management proposed a “one park, two zones” model. In current national park management policies, areas with minimal human interference and high levels of naturalness are designated as core protected areas. The remaining areas are classified as general control zones, intended to provide space for the production and living activities of indigenous inhabitants. However, similar to the situation in most national parks of developing countries, zoning remains largely a formality, lacking adequate policies for post-zoning management and clear institutional frameworks.^37^^,^^67^^,^^97^^,^^98^

Here, we analyze the logic of zoning management for national parks in developing countries, integrating insights from multi-theoretical perspectives to unite ecological protection and resource utilization. We construct a theoretical framework for zoning management of national parks based on the concept of “risk-value,” aiming to incorporate considerations of biodiversity, equity, and well-being goals into national park management. In addition to theoretical analysis, we also focus on management and the implementation of the framework. We take the Baishanzu National Park in China as an example to carry out an empirical study. By quantifying the level of resistance to ecological risk in the core protected area, as well as the economic value of ecological resources in the general control area, we categorized the Baishanzu National Park into 4 zones: CHR (core protected area—high ecological risk), CLR (core protected area—low ecological risk), GHE (general control area—high ecosystem service value), and GLE (general control area—low ecosystem service value). The result of the study shows that these 4 zones accounted for 8.92%, 43.03%, 14.23%, and 33.82%. Combining the theoretical analysis and zoning results, we propose the differentiated policies for the application of this framework in the Baishanzu National Park in China, which is of great significance for the construction of zoning management system of national parks in developing countries.

## Results

### Methods summary

We introduce a theoretical framework based on “risk-value” for zoning management in national parks in developing countries, applied empirically to Baishanzu National Park in China. This framework divides national parks into core protected and general control areas, aiming to balance ecological conservation and resource utilization. The distinct needs of these areas have led to the development of “risk-value” strategies: a “risk” strategy for the core protected area focuses on risk prevention and recovery, while a “value” strategy for the general control area emphasizes exchanging ecological and economic values to promote conservation efforts. To formulate differentiated strategies more scientifically, we employ two quantitative methods: the ERI quantifies ecological risk for the core protected area, evaluating ecosystem stability under environmental pressures,^71^^,^^72^^,^^73^^,^^74^^,^^75^^,^^76^^,^^77^ and the ESVs quantify the economic potential of ecological services for the general control area.^80^^,^^81^^,^^82^^,^^83^^,^^84^ These methods are validated in land use planning and ecological security management, supported by ArcGIS 10.7 for data analysis, facilitating scientifically informed policy development.^90^^,^^91^^,^^92^^,^^93^^,^^94^^,^^95^^,^^96^^,^^97^

### Multi-theoretical analysis of zoning management for national parks

National park issues, goals, and priorities are evolving with the changing social-ecological systems. Due to a combination of factors including diverse environmental issues to be solved and variations in institutional constrains for policy implementation, environmental policies developed by developing countries often differ from those commonly observed in developed countries.^99^ In the management of national parks in developing countries, several key points emerge. Firstly, national parks typically represent extensive, contiguous natural ecosystems endowed with a diverse array of resources, thus exhibiting spatial variations in ecological services. Secondly, the presence of indigenous communities within most parks complicates matters, as blanket protection measures across the entire park area may entail substantial economic costs and pose challenges in the equitable resettlement of these communities. Lastly, in the context of developing countries, national parks often contend with higher population densities and limited management funding, further exacerbating management challenges. In impoverished areas, where natural resource extraction frequently serves as the primary income source for local populations, conflicts may arise between conservation efforts and poverty alleviation goals.^68^^,^^100^^,^^101^ Therefore, we believe that the management of national parks in developing countries has to be more balanced between ecological protection and resource utilization. We not only consider biodiversity and ecological conservation but also explore the importance of fairness, justice, and human well-being in the context of national park management. To effectively address the diverse needs of stakeholders and the dynamic socio-ecological processes, the management of national parks should leverage multiple complementary environmental governance insights.^102^ We aim to explore the management logic of national parks in developing countries from a multi-theoretical perspective, encompassing various theoretical frameworks to provide a comprehensive understanding.

The common pool resources theory highlights a range of institutional structures, often created or collaboratively developed by resource users, which prove to be more effective in balancing diverse priorities compared to monocentric, state institutions.^103^^,^^104^ The polycentricity theory proposes to enhance the effectiveness of multiple organizations by establishing clear boundaries of responsibility and authority in decision-making processes, thereby demonstrating greater adaptive capacity.^105^^,^^106^^,^^107^ The adaptive co-management theory proposes a governance system that is flexible, collaborative, and learning-based, promoting management strategies that adapt to new information as it arises.^108^^,^^109^ These theories are guided by principles that emphasize the participation of various stakeholders, the sharing of power and responsibility, and the establishment of linkages between two or more spatial scales.^109^ The adaptive governance of common pool resources necessitates management strategies flexible enough to respond to changing social, economic, and ecological environments.^102^ As equity and justice in biodiversity conservation gained traction, the inclusive conservation theory emerged. The inclusive conservation theory builds on multiple theories, conceptualizes equity concerns in biodiversity and protected area management scholarship.^22^ It stresses the importance of considering biodiversity from diverse perspectives, where the input from both technical experts and local indigenous communities holds equal significance.^110^^,^^111^^,^^112^ In the context of national park management, more likely to achieve beneficial conservation outcomes are co-managed programs that involve indigenous peoples fully in biodiversity conservation, reduce economic inequalities, and sustain livelihood outcomes.^113^

Balancing different objectives often leads to conflicts, given the involvement of various stakeholders. In existing studies, zoning (the allocation of land units to specific uses) is a useful option for conflict mitigation and a key normative tool for managing protected areas.^30^ Spatial patterns strongly influence functional integrity by affecting critical ecological processes necessary for population persistence, biodiversity maintenance, and ecosystem integrity.^114^^,^^115^^,^^116^^,^^117^ Hence, the zoning methodology for national parks must be consistent with the ecological features of the landscape. Ecological function zoning is favored by many scholars and involves dividing an area into different ecological functional zones based on the analysis of ecosystem characteristics, ecosystem service patterns, and other ecological conditions, considering the spatial heterogeneity of the region.^97^^,^^118^^,^^119^^,^^120^ In the design of our national park management policies, we adopt the principle of ecological functional zoning. At the same time, we recognize that zoning policies, while achieving specific objectives, can lead to the “windfall-wipeout dilemma.” In this dilemma, stakeholders in areas zoned to allow human activities receive benefits not due to their own efforts, whereas owners of land in core conservation areas with restricted development face potential losses. This results in stakeholders in activity-permitted zones gaining from collective efforts, while those in strictly protected zones endure potential disadvantages.^121^^,^^122^ While the existing zoning management programs for national parks in developing countries are often mandatory and inflexible, our goal is to develop a more equitable and flexible zoning control strategy for these parks. The policy tool of Transfer of Development Rights (TDR) offers advantages over strict zoning regulations. It separates the right to develop land from a parcel designated as a sending area and transfers it to another parcel defined as a receiving area, thereby allowing for greater development intensity in the latter. After the sale or transfer of TDR, the sending area typically faces strict development restrictions.^123^ Our zoning management program for national parks in conjunction with the TDR entails assigning different objectives to distinct zones within the park, aiming to achieve environmental protection goals cost-effectively, while also generating certain economic value.

### The “risk-value” based theoretical framework of zoning management for national parks

**Figure 2:**
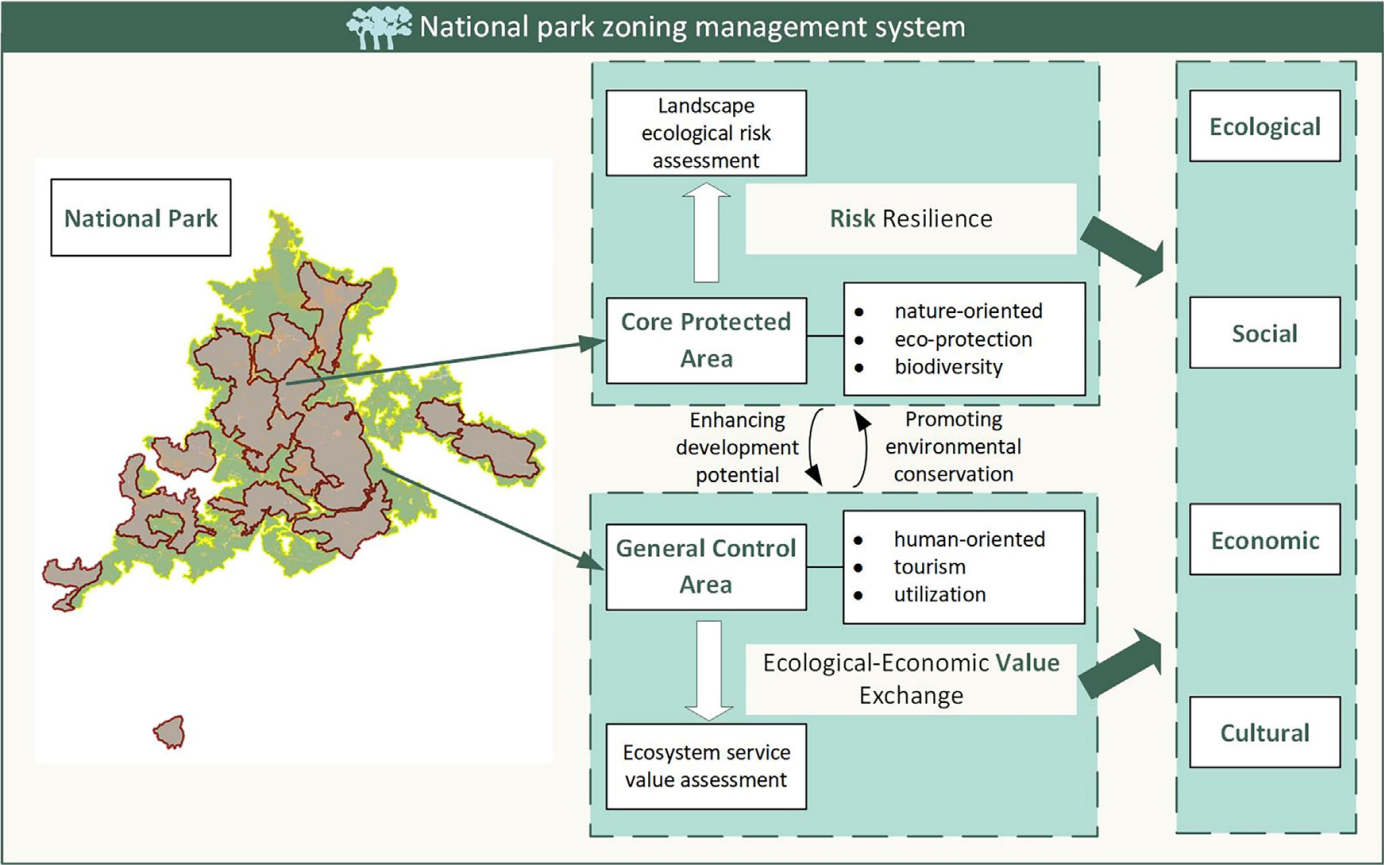
The theoretical framework of zoning management for national parks The maps were generated by ArcGIS 10.7 and Microsoft PowerPoint.

We provide a theoretical framework of the zoning management for national parks based on “risk-value” for developing countries (Figure 2). National parks in developing countries should serve as a type of protected area capable of effectively balancing the conservation of resources with the enhancement of human well-being, their functions mainly include two aspects: ecological protection and economic income. Of these, ecological protection should always come first, and economic income is typically achieved through the exchange of value between ecology and the economy. While these standards are broadly supported, their operationalization in developing countries is not straightforward. It is thus timely to consider how national park management can widen its frame to include biodiversity conservation, equity, and well-being goals. Hence, our framework centers on zoning management from a multi-theoretical perspective. It focuses on the two dimensions of ecological protection and resource utilization, aiming to achieve the goal of unifying ecological, social, economic, and cultural values. This framework initiates by delineating the national park into two distinct areas: the core protection area and the general control area. Subsequently, our attention is directed toward risk prevention and resilience strategies tailored for the core protected area, alongside ecological value conversion approaches aimed at the general control area. In this framework, we designate the core protected area as the sending area for development rights, and the general control area, as well as regions outside the national park, as the receiving areas. The TDR eliminates the land value increment resulting from non-individual efforts, while providing compensation to landowners with restricted development. This approach ensures that the benefits arising from non-individual efforts under zoning control are fairly distributed among stakeholders.^124^ Furthermore, the core conservation areas, rich in biodiversity and natural integrity, often require substantial protection costs, primarily funded by government expenditures, and ultimately borne by taxpayers. Through the transfer of development rights, the land in the sending areas is preserved, and a portion of the implementation costs is covered by the revenues generated from ecological tourism and other industries in the receiving areas.^125^ This framework allows national parks to meet specific objectives without imposing significant cost pressures. By utilizing different quantitative methods and differentiated policies, it fosters a positive cycle and mutual development between the designated zones. The goal is to create a win-win situation for biodiversity conservation, equity, and human well-being in developing countries.

The core protected area is mainly an uninhabited area within the national park, with strong biodiversity, high ecological sensitivity and high ecological protection value. The management responsibility for this area should lie with the government, which should implement the strictest protection policies. At the same time, considering the continuity of the area, it is allowed to appropriately allocate a small part of the core protection area with low-intensity anthropogenic activities, and to carry out relocation of a small number of indigenous residents in the area within the scope of controllable economic costs. This study believes that the core protected area is the most critical area for national parks to maintain the natural ecosystems, and that the management of this area should focus on the enhancement of risk-resistant capacity. So, we introduced the ERI to quantify the level of risk resistance in the area. On the one hand, policies for this area are formulated based on the evaluation results to achieve the purpose of ecological protection in national parks. On the other hand, the realization of ecological protection objectives can promote the enhancement of ecological values and the development of park tourism.

The general control area, situated on the periphery of the national park, serves as a buffer zone surrounding the core protection region. Although biodiversity is comparatively lower in this zone than in the core area, it is designated for the livelihoods of indigenous residents and to cater to human requirements for education, research, and recreation within the park. We recognize that this area holds significant natural potential to develop appealing and competitive environmental tourism products. Therefore, this study believes that the general control area is an important area for national parks to fulfill the tourism value and revitalize the economic potential of ecological resources. It emphasizes the necessity of leveraging market forces to facilitate the exchange of value between ecology and the economy. But unlike general tourist attractions, the value of the general control area of the national park is not realized by the economy formed by ticket income, but by the value of intangible ecosystem services. Hence, we introduced the ESV (including provisioning, regulation, support, and cultural services) to measure the economic value of intangible ecological products. The assessment findings can serve as a foundation for the government to implement ecological compensation programs. By utilizing tourism revenue and governmental subsidies, indigenous communities can derive tangible economic gains, boosting their dedication to ecological preservation, and facilitating the achievement of conservation objectives.

### Case study: Theoretical framework application in the Baishanzu National Park

We implement this theoretical framework in the Baishanzu National Park in China, where we refine and expand upon its existing zoning model. Through quantitative zoning, we assess the ecological risk resilience of the core protected area and the potential ecosystem value of the general control area for this park separately, thereby laying the groundwork for tailored policies aimed at adaptive management.

#### Spatial distribution characteristics of the ERI in the core protected area

**Figure 3:**
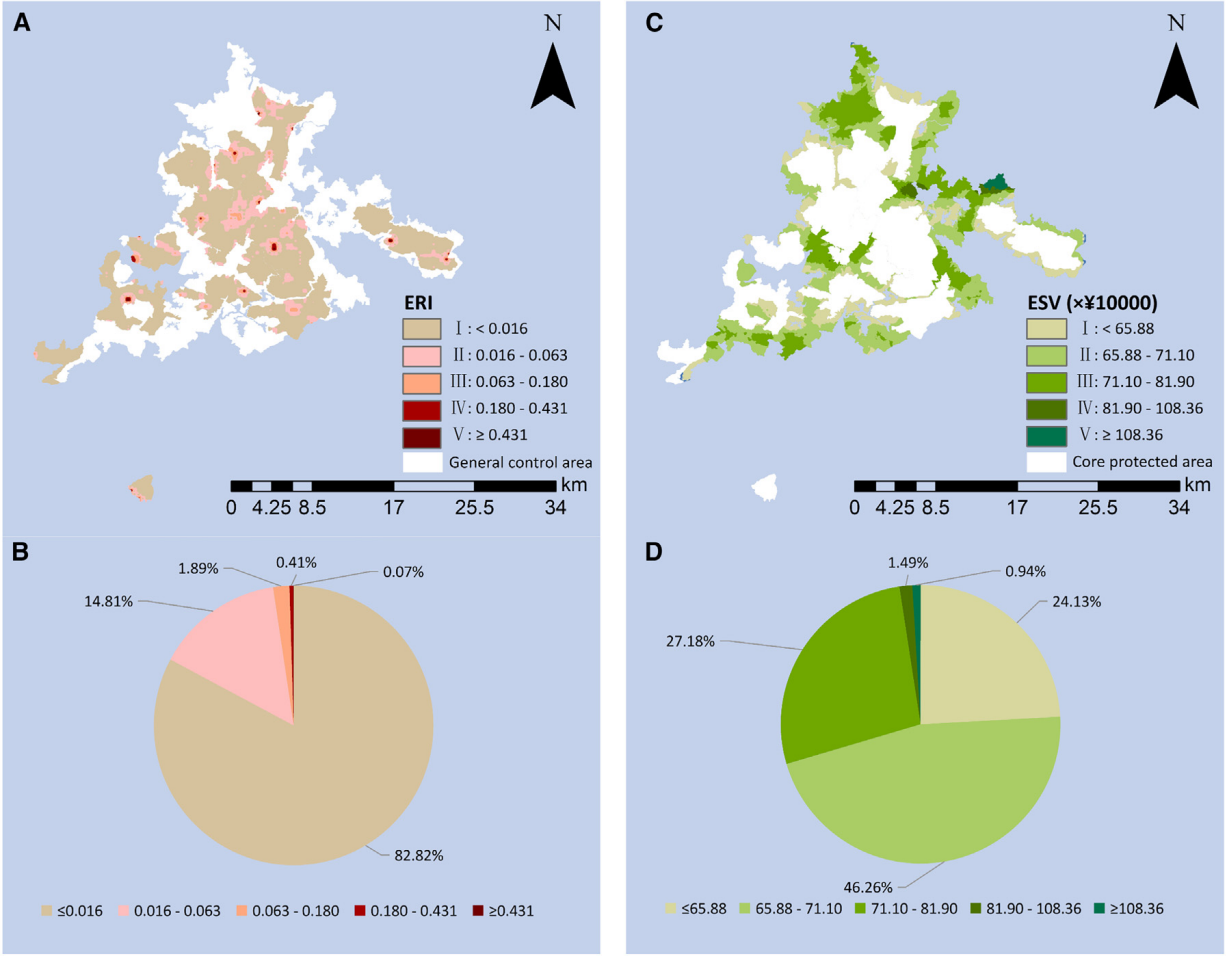
The result of the ERI and the ESV in the Baishanzu National Park (A) The spatial distribution of the ERI in the core protected area. (B) The spatial distribution of the ESV in the general control area. (C) The percentage of area of each ERI grade. (D) The percentage of area of each ESV grade. The maps were generated by ArcGIS 10.7.

Based on the grid model and the Kriging interpolation method performed with the geostatistical analyst module of ArcGIS software, the ERI of each risk assessment unit was spatially interpolated. In order to better discriminate the ecological risk in the areas, we used Jenks Natural Breaks method to categorize the results of the ERI into 5 grades (I: ERI＜0.016, II: 0.016≤ERI<0.063, III: 0.063≤ERI<0.180, IV: 0.180≤ERI<0.431, V: ERI≥0.431) to obtain the spatial distribution of the ERI in the Baishanzu National Park (Figure 3A). And we also get the results of the area percentage of each grade (Figure 3B). First, the area of grade I is the largest among these grades, accounting for 82.83%, with an area of 216.32 km^2^. The primary land use type in Grade I areas is arbor forest land, characterized by a concentrated and continuous spatial distribution with low patch fragmentation. The calculated ERI value is lower for these areas, indicating that they are less impacted by human activities and climate change, and have a stronger ability to withstand external disturbances.^126^ Second, the area percentage of II, III, IV, and V grades decreases successively, which are 14.81%, 1.89%, 0.41%, and 0.07%, respectively. Of these, grade V has the smallest area, only 0.18 km^2^. In terms of administrative divisions, these areas are mainly located at the border of Qingyuan County and Longquan County. The land types of grade V are mainly cultivated land, garden plot and bare land, and are a result of high ecological risk due to the high degree of fragmentation of the patches. This result reflects that these areas are less resilient to risk and that ecological protection should be strengthened. The overall analysis shows that the ecological risk value of the core protected area of the Baishanzu National Park is generally low, with the ERI below 0.016 in most areas, which reflects the park’s good control of the overall ecological risk of the core protected area. We attribute the good ecological status of the core protected area in the Baishanzu National Park to the fact that it has a large area of concentrated distribution of native evergreen broad-leaved forest zonal vegetation as well as many kinds of national key protection wildlife, with rich vegetation types, low patch fragmentation and high biodiversity.

#### Spatial distribution characteristics of the ESV in the general control area

To reveal the spatial characteristics of the ESV in the general control area, the Kriging interpolation method was used to map the distribution of ecosystem service value of the study area. According to the interpolation results, we used Jenks Natural Breaks method to categorize the results of the ESV into 5 grades (I: ESV＜65.88, II: 65.88≤ESV＜71.10, III: 71.10≤ESV<81.90, IV: 81.90≤ESV<108.36, V: ESV≥108.36) to obtain the spatial distribution of the ESV in the Baishanzu National Park (Figure 3C). And we also get the results of the area percentage of each grade (Figure 3D). First, the total ecosystem service value of the general control area was calculated to be RMB 1123.14 million. Second, the area of grade II is the largest among these grades, accounting for 46.26%, with an area of 111.75 km^2^. This is followed by grade III with a percentage of 27.18% and an area of 65.66 km^2^. Third, grade IV and V have the smallest percentage, 1.49% and 0.94%, respectively, with areas of 3.59 km^2^ and 2.28 km^2^. These areas are distributed in Jingning County, with the main land use types including water, forest land, and irrigated fields. Their high ecological value is due to the richness of the land use types, which can provide high cultural value and high ornamental value. The overall analysis shows that the ecological value of each grade in the general control area is more evenly distributed with small differences, and the overall ecological value is low with most of the evaluation units having an ecological value of less than RMB 819900.

We conducted an analysis of the ecosystem service value across different types within the general control area of Baishanzu National Park (Table 1). The findings reveal that regulating services hold the highest value, totaling RMB 883.75 million, representing 78.69% of the total value. This predominance is attributed to Baishanzu National Park’s characteristic forest ecosystem, where woodland dominates the land type. Extensive woodland and other ecological land contribute significantly to gas regulation, climate regulation, environmental purification, hydrological regulation, and soil retention. In contrast, the value of supporting services, provisioning services, and cultural services follows a declining order, with substantially lower numerical values compared to regulating services. Notably, cultural services exhibit the smallest value, accounting for only 4.52%. This can be attributed to the relatively homogeneous land type and limited cultural appreciation within the park. Furthermore, our field investigation identified a disconnect between the natural landscape of Baishanzu National Park and local culture and traditional industries. Insufficient brand awareness, tourism infrastructure, and related services hinder tourism development in the area. Overall, the ecosystem service value in the general control area of Baishanzu National Park exhibits significant variation, with regulating services dominating while other services remain underdeveloped. Future efforts should focus on enhancing the value of other ecological services, addressing the imbalance in their development without compromising the park’s primary conservation objectives. Specifically, cultural services represent a vital component in realizing the exchange of ecological and economic values of parks. Zoning management should leverage local cultural resources to foster related development, strengthen the cultural tourism sector, and integrate local primary and secondary industries such as agriculture and traditional handicrafts. This approach aims to establish a unique eco-tourism brand, enhance cultural services, and boost economic benefits, thereby supporting the conservation efforts in the core protected areas of the park.

**Table 1:** Value and percentage of various ecosystem service in the General Control Area of the Baishanzu National Park

Service Type	Value (RMB 10000)	Percentage (%)
Provisioning services	6391.74	5.69
Regulating services	88374.91	78.69
Supporting services	12472.44	11.10
Cultural services	5075.26	4.52

### Differentiated policies of zoning management for national parks

**Figure 4:**
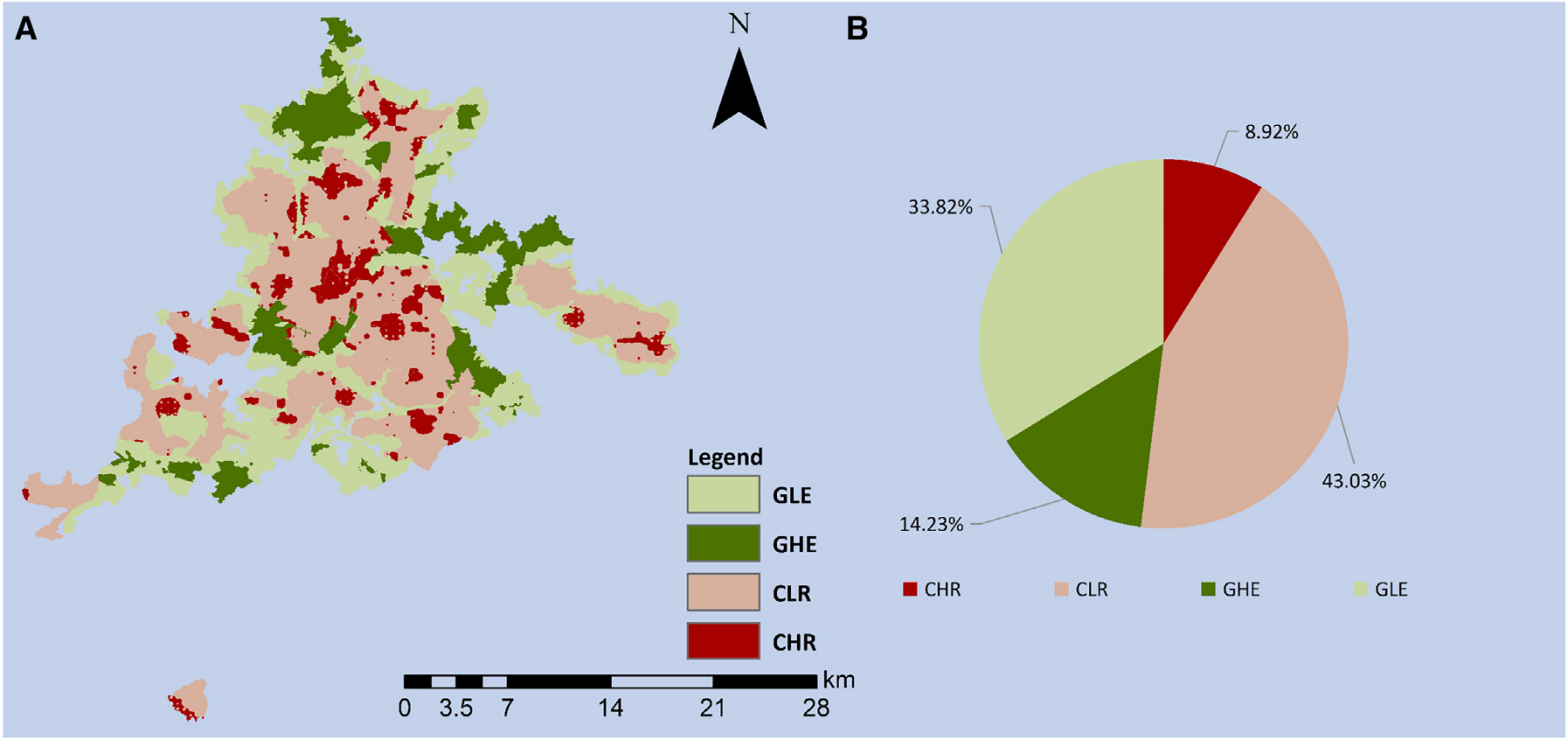
The zoning result for the Baishanzu National Park (A) The spatial distribution of the zoning result. (B) The percentage of area of each zone. The maps were generated by ArcGIS 10.7.

For the core protected area, we classify zones with the ERI greater than 0.016 as CHR (core protected area—high ecological risk) and those with the ERI less than 0.016 as CLR (core protected area—low ecological risk). For the general control area, we classify zones with the ESV greater than RMB 711000 as GHE (general control area—high ecosystem service value) and those with the ESV less than RMB 711000 as GLE (general control area—low ecosystem service value). Eventually, we categorized the Baishanzu National Park into 4 zones: CHR, CLR, GHE, and GLE (Figure 4A). The result of the study shows that these 4 zones accounted for 8.92%, 43.03%, 14.23%, and 33.82% (Figure 4B). We believe that a policy combining rigid and flexible management should be applied to national parks to promote a win-win situation for ecological protection and resource utilization. We propose the following differentiated policies of zoning management to address the different circumstances of these 4 zones.

For the CHR (core protected area—high ecological risk) zone. This zone is an area of high ecological risk in the core protected area of the national park, covering 44.85 km^2^. This zone is less risk-resistant and is a priority for ecological protection in national park. Thus, we advocate for a strict management policy for this area, designating it as the sending zone for development rights. The relevant management department should strengthen the control of this area, strictly protect the ecology of this area, and maximize the restriction of man-made activities in order to protect the integrity and originality of the natural ecosystem. In the future, the plant groups in the original habitat of the Baishanzu fir can be gradually restored through artificially assisted natural restoration, so as to further maintain the natural state of the ecosystems in the core protected area. Given its high ecological risk, the income generated from the receiving area of the development rights should be allocated to more than adequately compensate for the ecological protection costs in this zone.

For the CLR (core protected area—low ecological risk) zone. This zone is an area of low ecological risk in the core protected area of the national park, covering 216.32 km^2^. At present, this zone has a high capacity for ecological risk prevention and resistance, and ecological protection is effective. Therefore, in the management of this zone, it is essential to continue to maintain its good ecological condition. As this part of the area remains within the core protected area of the national park, we think it is equally important to apply a rigid policy to this zone. Obviously, anthropogenic activities are still not permitted in this zone. We suggest that the ecological risk of this zone should be regularly evaluated to prevent an increase in risk.

For the GHE (general control area—high ecosystem service value) zone. This zone is an area of high ecosystem service value in the general control area of the national park, covering 71.53 km^2^. This zone is an area of high ecosystem service value in the general control area of the national park, indicating that it has a high economic value of ecological products. A flexible policy could be adopted for this zone. Due to the good landscape appreciation of this zone, we consider that tourism can be suitably developed here. It should be necessary to fully explore the scientific, educational and tourism values of this area, such as combining with market-oriented operation to create a tourism brand, and increase the income of national parks to improve the living standards of local residents. The administration should be aware that this is the best zone to realize the utilization of the resources of the national parks. This area is used to generate income for the national park, stimulate economic benefits in neighboring regions, and compensate for the ecological protection costs incurred in the core conservation area.

For the GLE (general control area—low ecosystem service value) zone. This zone is an area of low ecosystem service value in the general control area of the national park, covering 502.73 km^2^. The ecosystem service value of this zone is low, and there is a certain amount of anthropogenic disturbance in it. Therefore, we propose that this zone should be used as the main area for local residents to engage in production and living, so as to solve the livelihood problem of the indigenous people. The administration should transfer as many residents as possible from other zones to this one while respecting their wishes and paying attention to their compensation. Additionally, we recommend transforming and upgrading the industries they are engaged in to minimize ecological damage caused by human activities. Training programs should be organized to enhance technical skills, enabling local residents to gain tangible economic benefits from participating in activities, such as ecological protection, nature education, and ecotourism services in national parks. This approach aims to foster a sense of identity and belonging among local residents and surrounding communities toward the national park, thereby increasing their enthusiasm for ecological conservation.

## Discussion

Combining theoretical and empirical analyses, this study constructs a theoretical framework for zoning management in national parks based on “risk-value” in developing countries (Figure 2). And we empirically apply this framework in the Baishanzu National Park in China, obtaining the spatial distribution of ecological risk and ecosystem service value in the study area through quantitative calculations (Figure 3). Building upon this foundation, we delineate the national park into 4 distinct zones (Figure 4). Tailored to the varied functional requirements of each zone, we propose differentiated policies for applying this framework. According to existing regulations for national parks, areas with minimal human interference and high naturalness are designated as core protected areas. In these areas, development rights should be ceded, and strict development controls should be implemented. The remaining areas are designated as general control areas, intended for human production and living, and can serve as receiving areas for development rights. Based on the refined zoning results from our quantitative evaluation, we recommend a strategy that combines rigid and flexible management. This strategy aims to link ecological conservation within the national park with the socio-economic activities of the community, promoting joint development of the park and the local community. For areas within the core protection zone with high ecological risks, all human development activities should be prohibited. Additionally, artificial measures should be implemented to enhance patch connectivity and integrity, improving the region’s ability to resist ecological risks. Ecological compensation should also be intensified to subsidize the high protection costs of these areas. In areas within the core protection zone with lower ecological risks, future efforts should focus on risk monitoring to ensure that ecological risks do not increase. In areas with high ecosystem service value within the general control zone, a more flexible management strategy can be adopted. This includes moderate development to establish distinctive ecological tourism while encouraging scientific research and educational activities. Market-oriented operations can generate income for the national park and stimulate economic development in the surrounding regions, thereby covering the ecological protection costs of the core protection zone. For areas with lower ESV within the general control zone, these should be designated as the main zones for the livelihoods and resettlement of indigenous residents. This approach addresses the residents’ livelihood and settlement issues while promoting industrial transformation and upgrading to minimize environmental damage. The establishment of national parks has emerged as a global trend aimed at reconciling nature conservation with human economic activities.^44^^,^^56^ By analyzing the logic of national park management in China (a typical representative of developing countries), this work also provides valuable information for the advancement of the national park concept. Moreover, these findings can provide a pathway for integrating policies with optimal cost-effectiveness for other developing countries to fulfill the targets of the CBD to meet global SDGs.

### National parks’ relevance in developing countries

National parks, as protected areas, play a crucial role in achieving goals, such as natural resource conservation, equity, livelihoods, and global SDGs.^23^ However, studies indicate persistent issues in establishing national parks in developing countries. Poverty remains significant in these regions, and national parks often face funding shortages. Revenue generated by national parks is vital for ecological protection.^62^^,^^63^^,^^64^^,^^65^^,^^66^ Additionally, the attitude toward natural resources is critical for the development of national parks in these regions, where higher population density and human interference frequently lead to social conflicts among different interest groups within the parks.^44^^,^^56^^,^^67^^,^^68^ Based on our field investigations of Baishanzu National Park in China, its current zoning plan remains idealistic, posing practical implementation difficulties. Overall, balancing ecological conservation and social development is a major challenge in establishing national parks in developing countries. Drawing on the experiences of national park construction in developed countries, we adapt these strategies to fit the unique situations of developing countries. The challenges of environmental protection and poverty alleviation resonate across different socio-economic backgrounds, underscoring the broad applicability of effective national park management strategies. We hope our theoretical framework and differentiated policies will aid in better implementing national parks in developing countries, achieving a balance between ecological conservation and economic development. Our proposed strategies align with sustainable development principles, protecting unique natural environments, and providing habitats for endangered species, while also focusing on the potential of national parks to improve local livelihoods. We aim to promote local economic development through industries such as ecotourism, encouraging local residents to actively participate in ecological protection. By adopting a holistic management framework that balances ecological conservation with socio-economic development, national parks can become catalysts for positive ecological changes in developing countries. Our study emphasizes the role of national parks in balancing ecological conservation and economic development. Addressing environmental protection, poverty alleviation, and sustainable development, the active construction and management of national parks may become powerful means to pursue a resilient and equitable future.

### More consideration of human-nature harmony in national park management

In the administration of national parks in developing nations, striking a balance between ecological preservation and resource utilization is essential. Unlike parks in developed countries, those in developing regions contend with denser populations and limited financial resources, necessitating a strategic approach that integrates the goals of ecology, society, economy, and culture. This research introduces a cost-efficient management model specifically designed for national parks in developing countries, aiming to meet conservation needs while maximizing benefits for local communities. At the same time, using the transfer of development rights (TDR) as a policy tool, we apply different strategies to core protected and general control areas, allowing for flexible management. This approach promotes both ecological protection and economic returns in national parks. The cost of ecological protection, traditionally borne by the government, will instead be partially covered by income generated from ecotourism and other industries in areas where human activities are permitted. Our theoretical framework for national park management harmoniously integrates the concepts of wilderness conservation and recreational use, custom-tailored for developing countries with careful consideration of their unique national circumstances. Central to the concept of national parks is the preservation of nature for the public good, offering protected areas and recreational spaces for human enjoyment, while simultaneously mitigating unplanned resource exploitation.^49^ Among the existing concepts of national park construction, the wilderness concept emphasizes the segregation of nature and society, prioritizing the ecological preservation of vast natural expanses, while the recreational concept is centered around human services, catering to the needs, and interests of the population.^39^^,^^42^^,^^44^^,^^45^ For developing countries grappling with the complexities of balancing societal and environmental interests,^56^^,^^62^^,^^63^^,^^64^^,^^65^^,^^66^^,^^67^^,^^68^ adopting a singular wilderness or recreational concept for national park management can pose challenges. The crucial role of national parks in advancing SDGs has garnered recognition within the international community. Therefore, it is worthwhile to explore avenues through which developing countries can promote the establishment of national parks. The management framework outlined in this study profoundly acknowledges the symbiotic relationship between human development and natural resources. It aims to foster a win-win scenario wherein economic benefits and ecological integrity are upheld simultaneously, without compromise. We advocate for the integration of conservation efforts with socioeconomic welfare, prioritizing ecological protection and ensuring livelihood security for the local population. Our aim is to achieve holistic prosperity encompassing ecological, social, economic, and cultural dimensions through the judicious utilization of natural resources.

### Applying the “risk-value” approach to national park management

By categorizing national parks into the core protected area and the general control area, management can adopt a “risk-value” approach. Therefore, we have integrated the concept of adaptive management, complemented by assessments using ERI and ESV, to operationalize this theoretical framework within the context of Baishanzu National Park. To enhance risk prevention and resilience in the core area, the utilization of the ERI is recommended for evaluating ecological risks. Landscape ecological risk assessment places significant emphasis on spatial and temporal heterogeneity, and understanding this heterogeneity is crucial as it informs regional risk prevention decisions and enhances landscape management practices.^78^ In the general control zone, focusing on the conversion of ecological products into economic worth, the introduction of the ESV is proposed to assess the value of intangible ecological products. Ecosystem services exhibit diverse service types, non-equilibrium service relationships, and variable spatial distribution. Quantitative assessment of these services serves as a foundation for understanding trade-offs and synergies within ecosystems. And we can better inform management decisions and optimize resource allocation within the national park. The efficacy and practicality of ERI and ESV have been extensively proven, rendering them valuable tools for spatial delineation and the development of targeted strategies within national parks. These tools facilitate a nuanced understanding of ecosystem dynamics and support evidence-based decision-making. Besides, strategies for adaptive management should be included in the follow-up. As socio-economic conditions evolve, land use will also change to some extent, and in the future the relevant areas within the park should be regularly assessed in terms of ERI and ESV in order to adjust strategies to respond to dynamic environmental factors and social needs. By considering both ecological and socio-economic factors, national park management can achieve a balance between conservation goals and the sustainable use of natural resources. This comprehensive approach ensures a holistic management strategy that balances conservation objectives with socioeconomic considerations, contributing to the sustainable development of national parks in developing countries.

### More consideration of differentiation in policy design

Implementing differentiated policies tailored to specific zones within national parks can foster a symbiotic relationship, striking a harmonious balance between ecological preservation and livelihood enhancement. National parks, characterized by vast and cohesive natural landscapes, demonstrate spatial diversity in ecological functions. Zones delineated according to evaluation outcomes typically serve varied purposes, warranting tailored policy approaches to address individual functional requirements. These zones, categorized based on assessment findings, often fulfill unique roles encompassing resource conservation, educational endeavors, scientific investigations, and recreational activities. The zoning of national parks is not merely a spatial division but a strategic management tool aimed at optimizing the conservation of biodiversity while promoting sustainable use of natural resources. Each zone within a national park plays a crucial role in supporting ecosystem services, biodiversity conservation, and sustainable development initiatives. For instance, the core protected area often encompasses ecologically sensitive habitats and serve as sanctuaries for endangered species, requiring stringent conservation measures to preserve their ecological integrity. In contrast, the general control area may accommodate a range of activities such as sustainable agriculture, ecotourism, and scientific research, aiming to enhance community livelihoods while minimizing environmental impacts. Tailored policy approaches are imperative to address the unique functional requirements of each zone effectively. These policies should be developed through a participatory process involving key stakeholders, including local communities, indigenous groups, conservationists, policymakers, and researchers. By incorporating diverse perspectives and local knowledge, policymakers can design policies that are contextually relevant, socially acceptable, and environmentally sustainable. Furthermore, tailored policies should align with broader SDGs to ensure holistic and integrated management of national parks. By promoting ecologically sustainable livelihoods, enhancing environmental education and awareness, and fostering community engagement, national parks can contribute significantly to achieving multiple SDGs, including those related to biodiversity conservation, poverty alleviation, sustainable agriculture, and climate action.

### Limitations of the study

We acknowledge that there are limitations to our analysis. One of the problems is that our research focuses on providing a “value at risk” based management framework for national parks in developing countries, but quantitative evaluation methods have been less explored. In the empirical process, we opted for the ERI and ESV, both of which have been extensively applied in existing studies. The evaluation results can serve as a reference for delineating ecological functional zones and formulating differentiated policies for national parks. However, as our study did not primarily focus on assessing ecological risks and ESVs, we did not consider the interactive operations between the two, nor did we conduct an in-depth exploration of the evaluation methodology. The ecological status of different national parks varies, indicating a need to optimize the evaluation methods according to the specific conditions of each region. This optimization will lead to more accurate evaluation results, which can in turn inform policy formulation. Moreover, limitations also exist in our failure to analyze the various stakeholders in national park management in more detail. The management of national parks presents a complex challenge, given that they are tasked with fulfilling multiple, sometimes conflicting objectives, such as biodiversity conservation, supporting local livelihoods, and promoting tourism.^102^ National parks exhibit variability across countries concerning land ownership, management authority, and resource utilization. They involve multiple stakeholders, including government entities, private individuals, businesses, and environmental organizations, each contributing to their management and administration. In the future, the mechanisms and theories of national park management can be analyzed in greater depth by examining the behavior of various stakeholders involved in the management process. Finally, we expect this study to offer valuable lessons for other developing countries. However, there are limitations due to the variations in institutional and social contexts across different countries. Therefore, a more localized approach to research and policy development should be adopted in specific studies.

## Resource availability

### Lead contact

Further information and requests for resources and reagents should be directed to and will be fulfilled by the lead contact, Yuzhe Wu (wuyuzhe@zju.edu.cn).

### Materials availability

No new materials were generated by this work.

### Data and code availability

• The existing, publicly available data are listed in the key resources table. The land use datasets obtained in this study are available upon reasonable request from the lead contact.
• This study does not report original code.
• Any additional information required to reanalyze the data reported in this study is available from the lead contact upon request.

## Acknowledgments

This work was financially supported by The National Key Research and Development Plan Project of China (2022YFC3800800), The Baishanzu National Park Scientific Research Project (2022JBGS05) and The National Natural Science Foundation of China (71874155).

## Author contributions

Conceptualization, Y.C. and Y.W.; methodology, Y.C. and Y.W.; formal analysis, Y.C. and Y.Z.; investigation, Y.C. and Y.W.; resources, Y.W.; data curation, Y.C.; writing—original draft, Y.C. and Y.Z.; writing—review and editing, Y.C., Y.Z., and Y.W.; visualization, Y.C. and Y.Z.; supervision, Y.Z. and Y.W.; project administration, Y.W.; funding acquisition, Y.W.

## Declaration of interests

The authors declare no competing interests.

## STAR★Methods

### Key resources table

**Table:** 

REAGENT or RESOURCE	SOURCE	IDENTIFIER
**Deposited data**		

Landsat 8 OLI images	Geospatial Data cloud	https://www.gscloud.cn/
Statistical data	The Bureau of Statistics of Lishui	http://tjj.lishui.gov.cn/

**Software and algorithms**		

ArcGIS 10.7	ESRI	https://www.arcgis.com/index.html

### Experimental model and study participant details

This study does not involve the use of experimental models or participants.

### Method details

#### Study area and data processing

Baishanzu National Park (118°57′49″E − 119°22′9″E, 27°32′25″*N* - 27°58′28″N) is located in the south of Lishui, Zhejiang Province, in the border area of Longquan, Qingyuan and Jingning counties, and its scope involves 5 nature reserves (Figure 1). The region in which it is located belongs to the middle-subtropical forest ecosystem, rich in biological resources, with diverse vegetation types and a high concentration of rare and endangered species. There are many research stations within it, which have important scientific research value. Also, it has a long history and culture, rich in folk culture, with high cultural value.

At the early stage of the establishment of this national park, the park structure of "one park and two zones" was proposed, with a total planning area of 502 km^2^, with the core protected area accounting for 51.82% and the general control area accounting for 48.18% (Figure 1D). This national park is one of the rare near-natural ecosystems in the economically developed regions of eastern China, and in the process of planning and management, it is necessary to take into account both ecological protection benefits and economic benefits. In view of the practical needs of ecological protection and ecological value conversion, it is of strong practical significance to choose this study area to conduct empirical research on the zoning management of national parks.

We preprocessed Landsat 8 OLI (Operational Land Imager) images of the study area from 2021 using ENVI 5.3 and ArcGIS 10.7, performing geometric correction, band fusion, mosaicking, cropping, atmospheric correction, and supervised classification to initially classify land use into 9 types: forest land, shrubland, field, garden plot, grassland, wetland, water, building land, and bare land. To extract the actual surface status of the study region more accurately, we referred to actual survey results and data provided by local officials, and we further subdivided forested and field and categorized land use in more detail into 12 types: arbor forest land, bamboo forest land, shrubland, other forest land, irrigated field, non-irrigated field, garden plot, grassland, wetland, water, building land and bare land (Figure 1C). The overall accuracy is more than 85%, and the kappa coefficient is greater than 0.75, which could meet the requirements of landscape research generally. Related studies have shown that the landscape sample area should be 2–5 times the average value to fully reflect the information of the landscape pattern around the sampling site.^96^^,^^127^^,^^128^ Therefore, we divided the study area into plenty of 400 m × 400 m grids totaling 3662.

#### Landscape ecological risk assessment

Ecological risk assessment is a scientific methodology used to evaluate the likelihood and consequences of potential adverse impacts resulting from human activities and climate change.^126^ Land use/cover-based landscape ecological risk assessment has been widely employed to describe the effects of anthropogenic disturbances or natural changes on the landscape’s composition, structure, functions, and processes within a given region.^129^ This method allows for a comprehensive assessment of multiple risk factors in the area. The Ecological Risk Index (ERI) utilized in this study combines the ecological vulnerability index and the landscape disturbance degree Index to comprehensively evaluate the landscape ecological risk of the region.^79^^,^^108^^,^^110^ This assessment considers landscape fragmentation, isolation, and dominance. A higher calculated index indicates greater ecological risk, suggesting more significant potential negative impacts from human activities and climate change, and a reduced ability to cope with external disturbances. The specific calculation formula is as follows:

(Equation 1) $${\text{ERI}}_{k}=\sum_{i=1}^{n}\frac{A_{ki}}{A_{k}}R_{i}$$

where *ERI*_*k*_ is the ecological risk index value of the kth risk assessment unit, *A*_*k*_ is the area of the kth risk assessment unit, *A*_*ki*_ is the area of ith LULC type in the kth risk assessment unit, *R*_*i*_ is the landscape’s loss index of ith LULC type. *R*_*i*_ is calculated as:

(Equation 2) $$R_{i}=F_{i}\times S_{i}$$

On the right side of the Equation 2, *F*_*i*_ is the ecological vulnerability index, with smaller values representing less ecological vulnerability. We set *F*_*i*_ values of the 12 LULC types are: 1 for building land, 2 for arbor forest land, 3 for bamboo forest land, 4 for shrubland, 5 for other forest land, 6 for garden plot, 7 for grassland, 8 for non-irrigated field, 9 for irrigated field, 10 for wetland, 11 for water, 12 for bare land.^93^^,^^126^^,^^130^^,^^131^^,^^132^ *S*_*i*_ is the landscape disturbance degree index of ith LULC type, which is calculated as:

(Equation 3) $$S_{i}=aC_{i}+bN_{i}+cD_{i}$$

On the right side of the Equation 3, *C*_*i*_ is the landscape fragmentation, *N*_*i*_ represents the landscape isolation, *D*_*i*_ is the landscape dominance index, *a*, *b*, and *c* reflect the effects of human disturbances on ecosystem and represents the weights of *C*_*i*_, *N*_*i*_ and *D*_*i*_, respectively, and we assigned values of 0.6, 0.3 and 0.1^132^*, C*_*i*_, *N*_*i*_ and *D*_*i*_ are calculated as:

(Equation 4) $$C_{i}=\frac{n_{i}}{A_{i}}$$

(Equation 5) $$N_{i}=\frac{A}{2A_{i}}\sqrt{\frac{n_{i}}{A}}$$

(Equation 6) $$D_{i}=\frac{M_{i}+L_{i}}{2}$$

where *n*_*i*_ is the number of patches in the ith LULC type, *A*_*i*_ is the area of ith LULC type, *A* is the total area of the core protected area, *M*_*i*_ is the ratio of the number of the patch of ith LULC type to the total number of all patches, *L*_*i*_ is the ratio of the area of ith LULC type to the total area of the core protected area.

#### Ecosystem service value assessment

We introduced ecosystem service values (ESV) to measure the economic value of intangible ecological products.^94^^,^^95^^,^^96^^,^^97^ For ease of calculation, we further categorized the LULC types into forest land, irrigated field, non-irrigated field, grassland, wetland, water, building land and bare land for ecosystem services valuation. According to the ecosystem service value equivalence coefficients^94^ and China’s equivalents of ecosystem service value supplied by per unit area of an ecosystem,^90^^,^^133^ we proposed equivalents of ecosystem service value supplied by per unit area of ecosystem for the Baishanzu National Park (Table S1), and the calculation equation of ecosystem services value is expressed as follows:

(Equation 7) $${ESV}_{k}=\sum_{i=1}^{n}A_{ki}\times {VC}_{i}$$

where *ESV*_*k*_ represents the ecosystem service value of the kth assessment unit, *A*_*ki*_ is the area of ith LULC type in the kth assessment unit, *VC*_*i*_ refers to the ecosystem service value per unit area of ith LULC type. Ecological value per unit equivalent factor equal to 1/7th of average food price. Based on the data published by the local government, the ecological service value per unit equivalent factor corresponding to the study area was calculated to be 231427 yuan/km^2.^

### Quantification and statistical analysis

All regression analyses were conducted using Microsoft Excel. Figures 1, 2, 3, and 4 were generated in ArcGIS 10.7, while Figure 2 was created using Microsoft PowerPoint.
