# Supplementary material for: Theoretical framework and differentiated policies for national park zoning management: A Baishanzu case study in China
Source: iScience. 2024 Nov 13;27(12):111377. doi: 10.1016/j.isci.2024.111377 (PMC11617969; doi:10.1016/j.isci.2024.111377)
Supplement: Document S1. Table S1 [file mmc1.pdf]

iScience, Volume *27*

Supplemental information

Theoretical framework and differentiated policies for national park zoning management: A Baishanzu case study in China

Yuchao Cai, Yingnan Zhang, and Yuzhe Wu

Supplemental table

Table S1. Equivalents of ecosystem service value supplied by per unit area of ecosystem for the Baishanzu National Park, Related to STAR Methods

Service Type	Forest Land	Irrigated Field	Non-irrigated Field	Grassland	Water	Building Land	Bare Land
**Provisioning services**							
Food production	0.25	1.36	0.85	0.23	0.8	0	0
Raw material production	0.58	0.09	0.40	0.34	0.23	0	0
Water supply	0.3	-2.63	0.02	0.19	8.29	0	0
**Regulating services**							
Gas regulation	1.91	1.11	0.67	1.21	0.77	0	0.02
Climate regulation	5.71	0.57	0.36	3.19	2.29	0	0
Purify environment	1.67	0.17	0.10	1.05	5.55	0	0.10
Hydrological regulation	3.74	2.72	0.27	2.34	102.24	0	0.03
**Supporting services**							
Soil retention	2.32	0.01	1.03	1.47	0.93	0	0.02
Nutrient cycling	0.18	0.19	0.12	0.11	0.07	0	0
Biodiversity conservation	2.12	0.21	0.13	1.34	2.55	0	0.03
**Cultural services**							
Aesthetic landscape	0.93	0.09	0.06	0.59	1.89	0	0.01
